# Modular Brain Network Organization Predicts Response to Cognitive Training in Older Adults

**DOI:** 10.1371/journal.pone.0169015

**Published:** 2016-12-22

**Authors:** Courtney L. Gallen, Pauline L. Baniqued, Sandra B. Chapman, Sina Aslan, Molly Keebler, Nyaz Didehbani, Mark D’Esposito

**Affiliations:** 1 Helen Wills Neuroscience Institute, University of California, Berkeley, Berkeley CA, United States of America; 2 Center for BrainHealth®, The University of Texas at Dallas, Dallas TX, United States of America; 3 Department of Psychology, University of California, Berkeley, Berkeley CA, United States of America; University of Texas at Austin, UNITED STATES

## Abstract

Cognitive training interventions are a promising approach to mitigate cognitive deficits common in aging and, ultimately, to improve functioning in older adults. Baseline neural factors, such as properties of brain networks, may predict training outcomes and can be used to improve the effectiveness of interventions. Here, we investigated the relationship between baseline brain network modularity, a measure of the segregation of brain sub-networks, and training-related gains in cognition in older adults. We found that older adults with more segregated brain sub-networks (i.e., more modular networks) at baseline exhibited greater training improvements in the ability to synthesize complex information. Further, the relationship between modularity and training-related gains was more pronounced in sub-networks mediating “associative” functions compared with those involved in sensory-motor processing. These results suggest that assessments of brain networks can be used as a biomarker to guide the implementation of cognitive interventions and improve outcomes across individuals. More broadly, these findings also suggest that properties of brain networks may capture individual differences in learning and neuroplasticity.

**Trail Registration**: ClinicalTrials.gov, NCT#00977418

## Introduction

Aging is associated with declines in various cognitive functions, such as attention, cognitive control, and memory [1]. There is emerging evidence that characterization of large-scale brain network properties provides an important framework for understanding such complex behaviors [2,3]. Previous work has shown that brain networks exhibit a modular organization, such that they are comprised of sub-networks, or modules. The extent of segregation of brain network modules can be quantified with a modularity metric [4], where highly modular networks have many connections within modules and fewer connections to other modules. Previous studies examining changes in modularity with aging have shown that older adults have less modular structural and functional brain networks than young adults [5–8], particularly in sub-networks thought to mediate ‘associative’ functions, such as the fronto-parietal control and dorsal and ventral attention modules, compared to those involved in sensory-motor processing [9].

Growing evidence also suggests that cognitive training interventions can induce neural plasticity and improve aspects of cognition in older adults [10–14]. In particular, cognitive training is hypothesized to have pronounced effects on brain networks [15]. Previous aging studies have demonstrated that cognitive training changes the white matter architecture of the brain as assessed with diffusion tensor imaging (DTI) [16–18], suggesting that training can alter structural network connectivity in older adults. Cognitive interventions have also been shown to change the functional connectivity of brain sub-networks in older adults. For example, in a group of healthy older adults, we previously found that strategy-based gist reasoning training improved abstract thinking, concept formation, and other executive functions as well as altered functional connectivity of the default mode sub-network and an ‘executive’ [19] sub-network [16]. Others have also shown that cognitive training in older adults increases functional connectivity within the ‘salience’ [20] sub-network, in addition to default mode and ‘executive’ sub-networks [21].

Despite previous work showing that cognitive training can alter network connectivity in older adults, there has been little focus on identifying baseline neural factors that can predict training-related improvements in cognition. In a study with traumatic brain injury (TBI) patients, we found that brain network organization assessed at baseline predicted training-related cognitive gains. Specifically, individuals with higher baseline brain network modularity showed greater improvements on tests of executive functioning after goal-oriented attention self-regulation training [22]. These findings suggest that brain network modularity can be used as a biomarker to guide cognitive interventions as well as provide insight into the neural mechanisms underlying these interventions. However, the utility of brain network modularity as a predictor of training outcomes has not yet been tested in healthy individuals.

In the present study, we examined the relationship between baseline brain network modularity and cognitive improvements in healthy older adults who participated in a previously published cognitive training protocol [16]. We hypothesized that older adults with higher baseline modularity would show greater training-related gains compared to those with lower modularity. Further, as aging has been shown to particularly affect the functional connectivity of association cortex sub-networks [9], we also hypothesized that the predictive relationship between modularity and training gains would be more pronounced in association cortex sub-networks compared to sensory-motor sub-networks.

## Materials and Methods

### Subjects

Subjects from the original report (N = 37) were included in this study if they had structural MRI and resting-state fMRI data [16]. These 29 subjects were cognitively normal older adults (age range: 57–70) that had been randomized to a wait-list control group (N = 14; mean ± SD age: 64.4 ± 3.3; 9 females; mean ± SD IQ: 120.4 ± 11.7) or cognitive training group (N = 15; mean ± SD age: 63.1 ± 2.9; 9 females; mean ± SD IQ: 122.1 ± 8.3). The groups were matched on age (t(27) = 1.13, p = 0.27), distribution of gender (Χ^2^(1, N = 29) = 0.06, p = 0.81), and WASI IQ (t(27) = -0.46, p = 0.65). Subjects were screened for dementia (Telephone Interview of Cognitive Status-Modified, TICS-M), early cognitive impairment (Montreal Cognitive Assessment, MoCA), and depressive symptoms (Beck Depression Inventory-II, BDI) and underwent a complete medical assessment by a physician to ensure good health. Criteria for inclusion were: right-handed, native English speaker, no history of neurological or psychiatric conditions, normal IQ range, normal cognitive status (TICS-M ≥ 28, MoCA ≥ 26, BDI ≤ 14), minimum of high school diploma, no MR scanning contraindications, and normal body mass (BMI ≤ 40). This study was approved by the ethical review boards of the University of Texas Southwestern Medical Center at Dallas, the University of Texas at Dallas, and the Cooper Institute. All subjects provided written informed consent before participating in the study.

### Cognitive training (SMART)

The cognitive training group engaged in 12 weeks of ‘Strategic Memory and Reasoning Training’ (SMART) [23]. SMART is a strategy-based gist reasoning training program that focuses on the use of strategic attention, integrated reasoning, and innovation to improve cognitive control processes related to gist reasoning. Each week, subjects completed an hour of small group training (N ≤ 5 in each group) and two one-hour sessions of home practice [16]. For home practice, subjects in the SMART group logged practice time and assignment completion, which was reviewed by the trainers to provide feedback on the subject’s performance. To examine cognitive changes specifically associated with SMART, these subjects were compared to a group of wait-list control subjects.

### Neurocognitive measures

A battery of neurocognitive measures was collected at three time points (‘baseline’ or pre-training, mid-training, and post-training) as described in Chapman et al. (2015). In this study, we focused on the pre- and post-training assessments of gist reasoning and concept abstraction, as measured by the Test of Strategic Learning (TOSL [23]) and WAIS-III Similarities [24], respectively. These measures were shown to improve following SMART in the full sample of subjects [16] and are considered to be near- and far-transfer effects related to SMART, respectively. Gist reasoning is a measure of complex abstraction and involves synthesizing abstracted meanings from lengthy textual information (e.g., global ideas conveyed in an article). Three randomized versions of the TOSL were administered at the three time points of assessment and prior work has reported the reliability and validity of this measure [25]. Concept abstraction is a measure of simple concept abstraction and involves identifying categorical abstraction between two items (e.g., how a fly and an airplane are alike). We subsequently refer to WAIS-III Similarities as ‘Similarities’.

### MRI acquisition and preprocessing

At baseline, MRI scans were collected with an 8-channel head coil on a 3T Philips scanner. A T1-weighted sequence was used to acquire anatomical images (in-plane resolution = 1 mm^2^, 160 1-mm thick sagittal slices, TR/TE = 8.3/3.8 ms). A T2*-weighted echoplanar imaging (EPI) sequence was used to acquire a four-minute resting-state scan (in-plane resolution = 3.44 mm^2^, 36 4-mm thick ascending axial slices, TR/TE = 2000/30 ms).

Standard preprocessing of MRI data was carried out with Configurable Pipeline for the Analysis of Connectomes (CPAC [26]). First, EPI data was slice-time and motion corrected and co-registered to the T1-weighted structural image. Then, signals from motion (Friston 24-parameter model [27]), the top five principal components from white matter and CSF voxels [28], and linear and quadratic trends were regressed out. Lastly, EPI data was bandpass filtered (0.009–0.08 Hz), scaled to a global 4D mean of 10000, and warped to MNI space.

### Functional connectivity and network analyses

Subjects’ T1-weighted anatomical scans were warped to MNI space and parcellated into 264 regions of interest (ROIs) [29]. Time-series from EPI data were averaged over the voxels in each ROI. Nine ROIs were excluded from subsequent analyses because they were missing coverage in at least one subject. Correlation matrices were created for each subject by correlating the time-series between each pair of ROIs using Pearson’s correlation coefficient and applying a Fisher z-transform. Adjacency matrices were created by thresholding each correlation matrix over a range of thresholds (the top 2–10% of connections in 2% increments), resulting in unweighted and undirected graphs comprised of nodes, or ROIs, and edges, or the connections between them. While this range of connection density thresholds is similar to that used in the creation of the Power et al. (2011) atlas and an approach we have taken previously [30], it should be noted that other thresholds may be equally valid (e.g., [31]). We then assigned each ROI to a module as defined in Power et al. (2011) and quantified each subject’s network modularity, defined as:

$$Modularity=\sum_{i=1}^{m}(e_{ii}-a_{i}^{2})$$

where *e*_*ii*_ is the fraction of connections that connect two nodes within module *i*, *a*_*i*_ is the fraction of connections connecting a node in module *i* to any other node, and *m* is the total number of modules in the network [4]. Modularity is a measure that compares the number of connections within modules to the number of connections between modules across the network. Modularity will be close to 1 if all connections fall within modules and it will be 0 if there are no more connections within modules than would be expected by chance. As there are multiple methods for grouping nodes into modules, we also repeated these analyses using spectral clustering [32] to confirm that our results could generalize across other clustering algorithms and were not driven by imposing the specific Power et al. (2011) module assignments across all subjects. Importantly, the spectral method groups ROIs into subject-specific modules to generate the modular organization with the highest modularity value for this algorithm. It should be noted, however, that exhaustively searching through all possible ROI groupings to identify the ‘true’ modular organization with the highest modularity value is a computationally intensive problem [33]. Spectral clustering is one commonly used heuristic used to approximate the organization with the highest modularity value. Unless otherwise noted, modularity values are presented as the average across connection density thresholds. Although we confirm that our results are similar across commonly used connection density thresholds and clustering algorithms, the optimal methods for uncovering modular network organization remain an open question [34].

As aging has been shown to have a more pronounced effect on the modularity of association cortex modules compared with sensory-motor modules [9], we also examined the differential contribution of the modularity of these sub-networks to predicting cognitive gains in older adults. As described previously, the whole-brain modularity metric is computed as the sum of the modularity values for each module [4]. Using the Power et al. (2011) module assignments, we computed the baseline modularity of each sub-network, or module. We then classified these modules as ‘sensory-motor’ or ‘association cortex’ according to the groupings described in Chan et al. (2014). Specifically, sensory-motor modules included the auditory, somatomotor (hand and mouth), and visual modules; association cortex modules included the cingulo-opercular, default mode, dorsal attention, fronto-parietal, salience, and ventral attention modules. To compute average baseline sensory-motor and association cortex modularity, we averaged the modularity values over the sub-networks, or modules, in each group.

Finally, as weaker network connections that do not pass our connection density thresholds may also be informative in predicting training outcomes, we quantified the ‘segregation’ [9] of each module from the Power et al. (2011) assignments, defined as:

$$Segregation=\frac{{\bar{Z}}_{w}-{\bar{Z}}_{b}}{{\bar{Z}}_{w}}$$

where ${\bar{Z}}_{w}$ is the average Fisher-transformed correlation between nodes belonging to the same module and ${\bar{Z}}_{b}$ is the average Fisher-transformed correlation between nodes belonging to that module and nodes in any other module [9]. Segregation was calculated for each module, where a segregation value greater than 0 reflects greater within- than between-module connectivity (i.e., greater module segregation) and a segregation value of 1 reflects no between-module connectivity. Importantly, this approach does not employ thresholding and binarization and, as such, takes all network connections and their weights into account. We first computed baseline whole-brain network segregation by averaging the segregation values over all modules. We next computed baseline sensory-motor and association cortex sub-network segregation by averaging the segregation values over the modules belonging to the sensory-motor and association cortex groups, respectively, as described above.

### Statistical analysis

To confirm that the effects of SMART on the TOSL and Similarities were similar to the original report [16] in this reduced sample of subjects, we first conducted repeated measures ANOVAs on these neurocognitive measures with a within-subjects factor of time (pre- and post-training) and a between subjects factor of group (SMART and Control). We report effect sizes for these ANOVAs as partial eta-squared (η2p).

To quantify the relationship between baseline whole-brain modularity and training-related cognitive gains, we examined the correlation between baseline modularity and cognitive gains on the TOSL and Similarities. Cognitive gains were computed as the difference in post-training and pre-training (or baseline) scores, separately in the Control and SMART groups. Due to the relatively small sample size in each group, we conducted non-parametric Spearman correlations to reduce influence from extreme values, unless we were examining partial correlations that controlled for variables of non-interest (e.g., baseline TOSL or in-scanner motion). We denote Spearman correlations as ‘rho’ and partial correlations as ‘r_p_’. We also report 95% bias-corrected and accelerated (BCa) confidence intervals (CIs) based on 2000 bootstrap samples for main correlation analyses. We compared the magnitude of correlations between Control and SMART groups [35], after converting Spearman’s correlation coefficients to Pearson’s correlation coefficients using the formula described by Myers and Sirois [36].

Finally, to assess the differential effects of sensory-motor and association cortex modularity on predicting training-related gains, we used two complementary approaches. First, we examined the relationship between cognitive gains and sensory-motor and association cortex modularity (i.e., modularity derived from the thresholded and binarized correlation matrices) in each group. Second, to confirm that the relationship between modularity and cognitive gains was similar when using all network connections and their weights, we examined the relationship between cognitive gains and sensory-motor and association cortex segregation in each group.

## Results

### Cognitive changes after SMART

**Fig 1:**
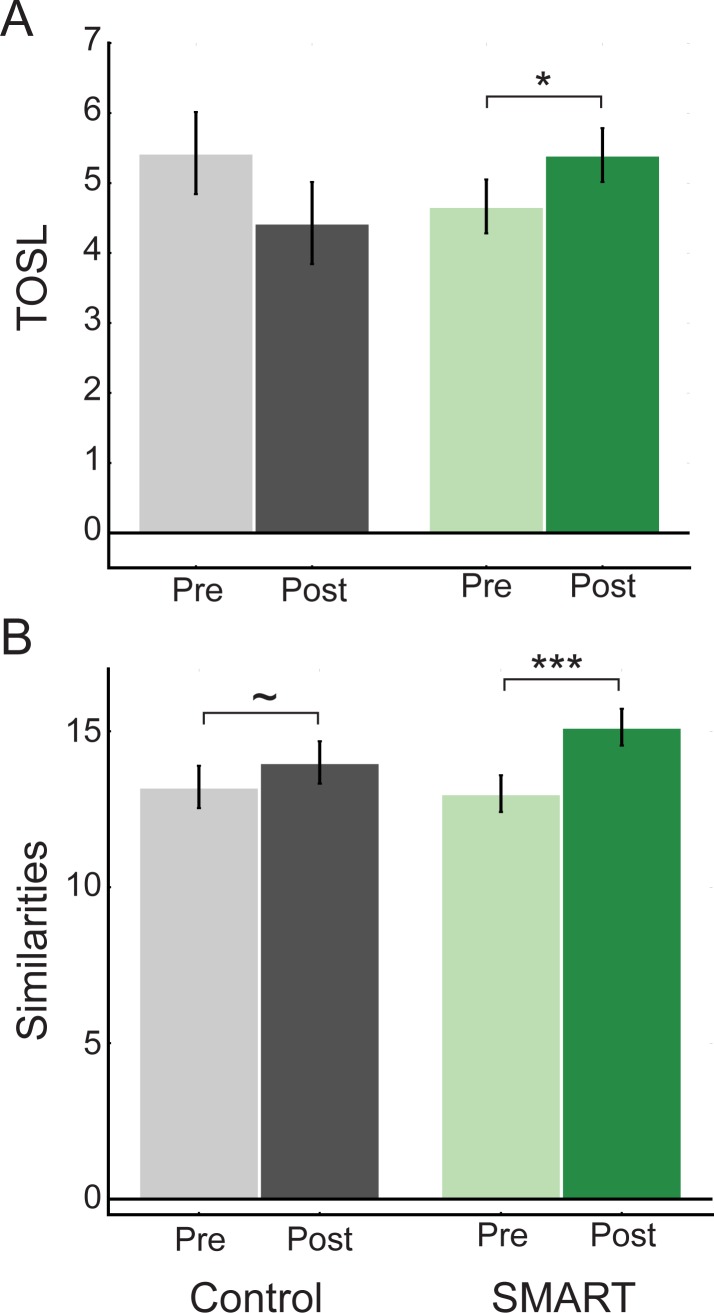
SMART-related cognitive changes. (A) Performance on the Test of Strategic Learning (TOSL) and (B) WAIS-III Similarities for Control and SMART groups pre- and post-training. Scores are presented as in Chapman et al. (2015), where the TOSL is presented as raw scores and Similarities is presented as standard scores. Data are presented as mean ± SEM. ~P ≤ 0.10, *P ≤ 0.05, ***P ≤ 0.001.

Similar to the larger dataset previously reported [16], there were no group differences in baseline performance on the TOSL (complex abstraction; t(27) = 1.06, p = 0.30) or Similarities (simple concept abstraction; t(27) = 0.23, p = 0.82). However, a significant group by time interaction for the TOSL (F(1,27) = 5.82, p = 0.02, η2p = 0.18) and Similarities (F(1,27) = 4.03, p = 0.06, η2p = 0.13) suggested that the groups exhibited different patterns of pre- and post-training performance. Within the SMART group, subjects improved on both the TOSL (Fig 1A; t(14) = -2.13, p = 0.05) and Similarities (Fig 1B; t(14) = -4.22, p = 0.001), while control subjects did not (t(13) = 1.55, p = 0.15; t(13) = -1.81, p = 0.09, respectively).

### Relationship between baseline whole-brain modularity and cognitive changes

**Fig 2:**
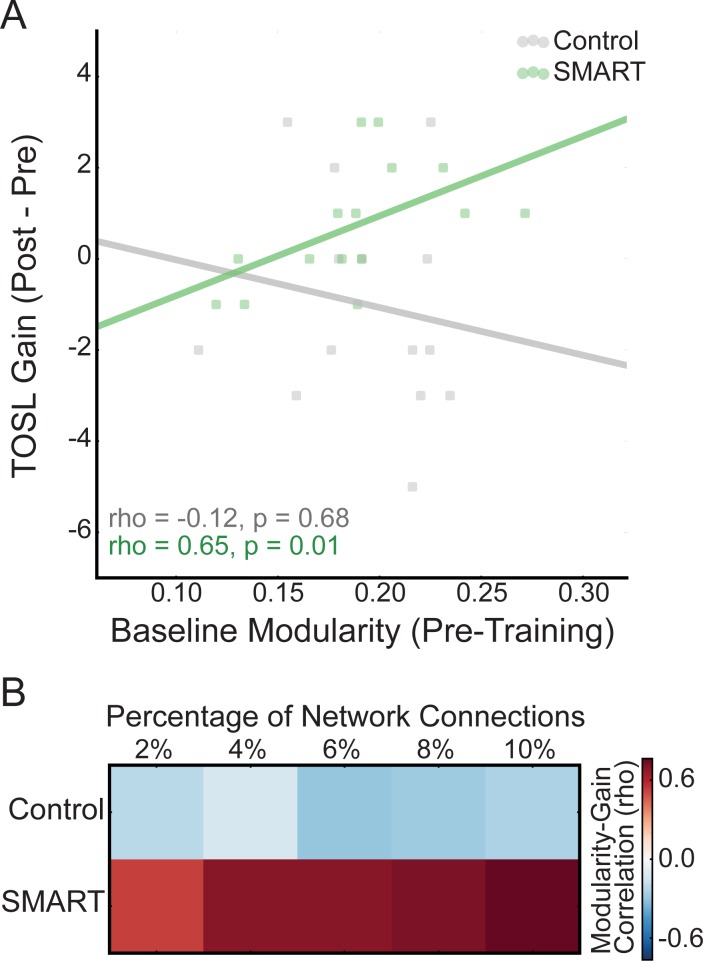
Relationship between baseline modularity and cognitive changes after SMART. (A) Relationship between baseline whole-brain modularity and change in performance on the TOSL, calculated as the difference of post-training and pre-training (i.e., ‘baseline’), in Control (grey) and SMART (green) groups. Here, modularity values were calculated for each connection density threshold and averaged for each subject. (B) Relationship between baseline modularity and change in performance on the TOSL for each connection density threshold in each group.

Baseline whole-brain modularity was similar in the SMART and Control groups (t(27) = 0.39, p = 0.70) and was not related to subject age in either group (Control: rho(12) = -0.29, p = 0.31; SMART: rho(13) = -0.14, p = 0.62). In the SMART group, baseline modularity was positively correlated with training-related gains in performance on the TOSL (Fig 2A; rho(13) = 0.65, p = 0.01, BCa 95% CI [0.35, 0.81]), but not on Similarities (rho(13) = 0.03, p = 0.90, BCa 95% CI [-0.52, 0.52]). In the Control group, there was no relationship between modularity and performance on either the TOSL (rho(12) = -0.12, p = 0.68, BCa 95% CI [-0.66, 0.50]) or Similarities (rho(12) = -0.26, p = 0.37, BCa 95% CI [-0.76, 0.35]). As the correlation between baseline modularity and training gains was not significant for Similarities, we focused on training-related gains on the TOSL for the remaining analyses.

The modularity-TOSL gain correlations were significantly different between the Control and SMART groups (Fig 2A; p = 0.03). Further, while baseline performance on the TOSL was negatively related to TOSL gains in both groups (Control: rho(12) = -0.83, p < 0.001; SMART: rho(13) = -0.80, p < 0.001), there was no relationship between baseline TOSL and modularity in either group (Control: rho(12) = 0.20, p = 0.49; SMART: rho(13) = -0.33, p = 0.24). We also confirmed that, when controlling for baseline TOSL performance, the modularity-TOSL gain relationship remained significant in the SMART group (r_p_(12) = 0.57, p = 0.03), but was not significant in the Control group (r_p_(11) = 0.36, p = 0.22). Finally, as previous studies have integrated network measures over connection density thresholds rather than averaging (e.g., [22]), we confirmed that integrated baseline modularity was correlated with training-related gains on the TOSL in the SMART but not Control groups (Control: rho(12) = -0.15, p = 0.60; SMART: rho(13) = 0.68, p = 0.01).

We took several steps to examine the robustness of our findings. First, to ensure that the relationship between modularity and cognitive gains was not differentially present across the connection density thresholds, we also examined the modularity-TOSL gain relationship for each threshold separately in the two groups. The correlations between modularity and the TOSL changes were consistent across the connection thresholds (Fig 2B). Specifically, across connection densities, baseline modularity was significantly related to TOSL changes in the SMART group (2% density: rho(13) = 0.53, p = 0.04; 4% density: rho(13) = 0.69, p = 0.004; 6% density: rho(13) = 0.70, p = 0.004; 8% density: rho(13) = 0.72, p = 0.002; 10% density: rho(13) = 0.76, p = 0.001), but not in the Control group (2% density: rho(12) = -0.21, p = 0.47; 4% density: rho(12) = -0.13, p = 0.66; 6% density: rho(12) = -0.30, p = 0.30; 8% density: rho(12) = -0.27, p = 0.34; 10% density: rho(12) = -0.24, p = 0.41). Further, we confirmed that there were no group differences in baseline modularity across connection thresholds (2% density: t(27) = 0.45, p = 0.65; 4% density: t(27) = 0.40, p = 0.70; 6% density: t(27) = 0.44, p = 0.67; 8% density: t(27) = 0.34, p = 0.74; 10% density: t(27) = 0.21, p = 0.84). Second, given that multiple methods for identifying network modules exist, we also identified subject-specific modules using a spectral clustering algorithm [32], instead of imposing the same modules as defined in Power et al. (2011) for all subjects. In each group, the correlations between modularity and training gains on the TOSL were similar when using the spectral method (Control: rho(12) = -0.38, p = 0.18; SMART: rho(13) = 0.76, p = 0.001). Finally, as in-scanner motion can spuriously affect functional connectivity estimates [37–39], we confirmed that motion (framewise displacement (FD) [37]) was similar between the groups (mean ± SD FD: Control: 0.16 ± 0.10; SMART: 0.15 ± 0.05; t(27) = 0.27, p = 0.80) and was not related to modularity in either group (Control: rho(12) = 0.06, p = 0.84; SMART: rho(13) = -0.40, p = 0.14). Further, controlling for motion did not substantially alter the modularity-TOSL training gain correlations (Control: r_p_(11) = -0.16, p = 0.60; SMART: r_p_(12) = 0.55, p = 0.04).

### Relationship between sensory-motor and association cortex modularity and cognitive changes

**Fig 3:**
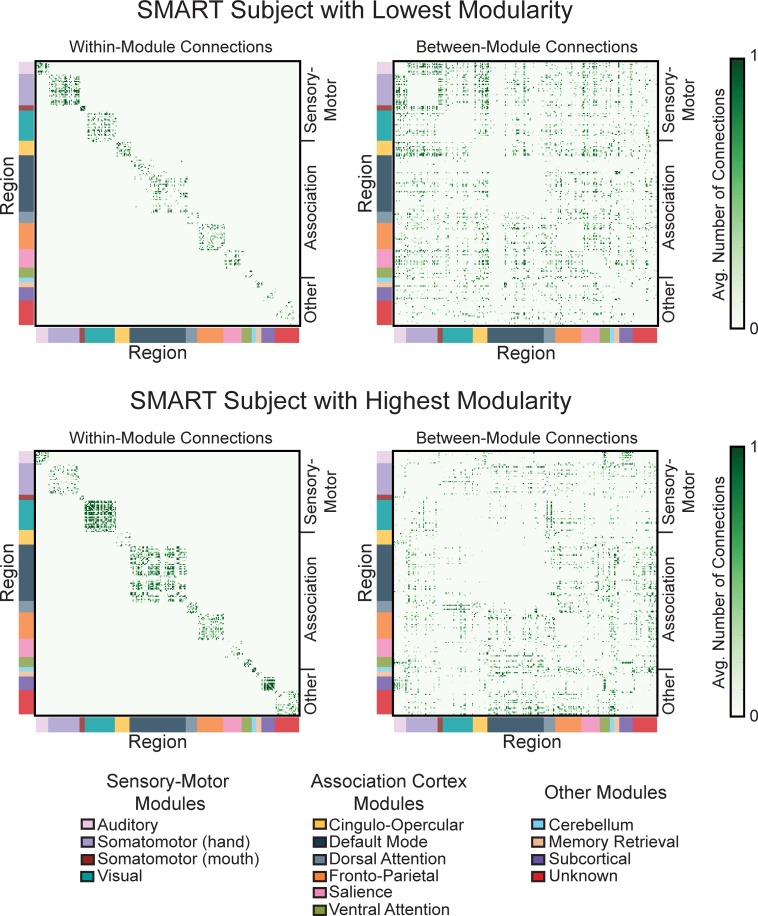
Module connectivity for SMART subjects with low and high modularity. Depictions of within- (left) and between- (right) module connections for SMART subjects with low (top) and high (bottom) brain network modularity. The presence or absence of a connection was calculated for each connection density threshold (i.e., an adjacency matrix) for the top 2–10% of connections in 2% increments. For illustration purposes, we then averaged the adjacency matrices over thresholds for each subject, where edges represent the proportion of thresholds for which a connection was present between two regions (ranging from 0 to 1). Brain regions are colored according to their module assignments in Power et al. (2011) and are grouped into sensory-motor and association cortex modules as defined in Chan et al. (2014). The subject with high modularity has many connections within modules and fewer connections between modules compared to the subject with low modularity.

Examination of the connectivity patterns for SMART subjects with the lowest and highest brain network modularity confirmed that more modular networks were characterized by many connections between regions belonging to the same module and relatively few connections between these modules (Fig 3). In the Control and SMART groups, the concentration of connections within and between modules varied across the sub-networks belonging to sensory-motor or association cortex modules. Comparisons of baseline sensory-motor and association cortex modularity in each group showed that modularity was greater in sensory-motor compared with association cortex sub-networks in both Control (t(13) = 8.52, p < 0.001) and SMART (t(14) = 5.71, p < 0.001) groups. Further, sensory-motor and association cortex modularity were not significantly correlated in either group (Control: rho(12) = -0.24, p = 0.42; SMART: rho(13) = 0.04, p = 0.90).

**Fig 4:**
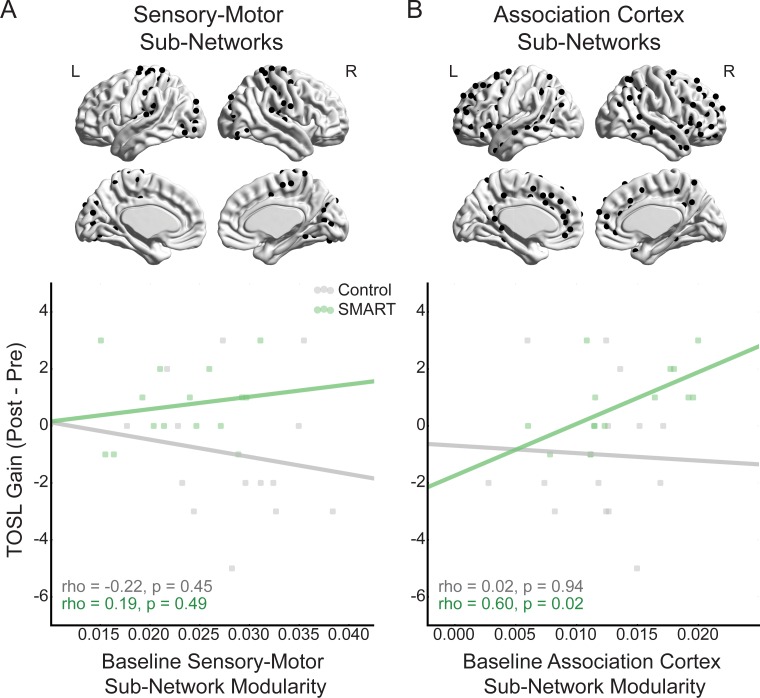
Relationship between sensory-motor and association cortex modularity and cognitive changes after SMART. Relationship between sensory-motor (A) and association cortex (B) sub-network modularity and TOSL gain in the Control (grey) and SMART (green) groups. Cerebral cortex regions belonging to sensory-motor and association cortex modules are plotted on sagittal views of the brain in A and B, respectively.

Given that the modularity of sensory-motor and association cortex sub-networks were not related, we next examined the differential contribution of the modularity of sensory-motor and association cortex sub-networks to the prediction of training-related TOSL changes. In the SMART group, the TOSL gain was significantly related to association cortex sub-network modularity (rho(13) = 0.60, p = 0.02, BCa 95% CI [0.07, 0.93]), but not sensory-motor sub-network modularity (rho(13) = 0.19, p = 0.49, BCa 95% CI [-0.47, 0.79]) (Fig 4). In the Control group, there was no relationship between either sensory-motor sub-network modularity (rho(12) = -0.22, p = 0.45, BCa 95% CI [-0.79, 0.50]) or association cortex sub-network modularity (rho(12) = 0.02, p = 0.94, BCa 95% CI [-0.47, 0.50]). To further examine how sensory-motor and association cortex sub-network modularity contributed to the whole-brain modularity-TOSL gain correlation in the SMART group, we conducted the whole-brain modularity and TOSL gain correlation controlling for either sensory-motor or association cortex sub-network modularity. Controlling for sensory-motor modularity did not substantially alter the relationship between whole-brain modularity and TOSL gain (r_p_(12) = 0.58, p = 0.03), while controlling for association cortex modularity reduced the relationship between whole-brain modularity and TOSL gain (r_p_(12) = 0.12, p = 0.69).

### Relationship between module segregation and cognitive changes

**Fig 5:**
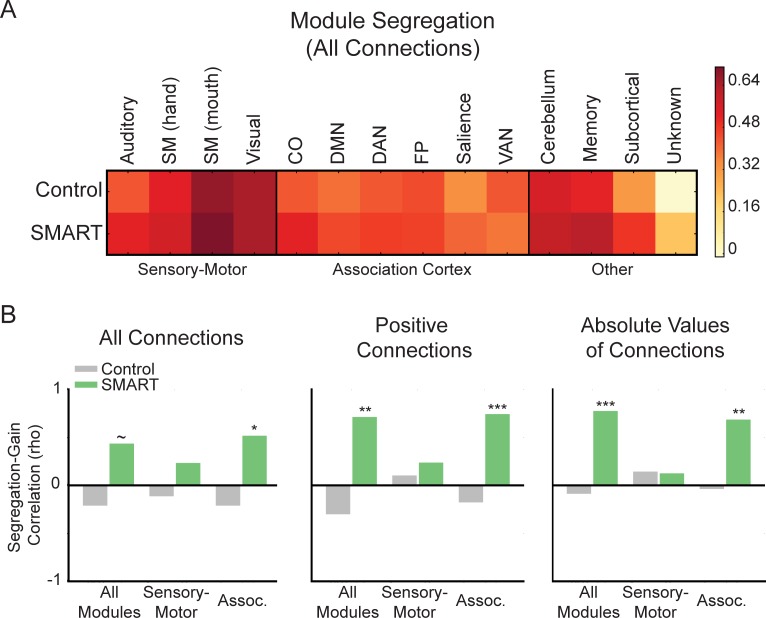
Relationship between module segregation and cognitive changes after SMART. (A) Module segregation for the Control and SMART groups. (B) Correlation between module segregation (all modules, sensory-motor modules, and association cortex modules) and change in performance on the TOSL. Correlations were performed using all network connections (left), only positive network connections (middle), and the absolute values of connections (right). SM, somatomotor; CO, cingulo-opercular; DMN, default mode network; DAN, dorsal attention network; FP, fronto-parietal, VAN, ventral attention network, Assoc., association cortex. ~P ≤ 0.10, *P ≤ 0.05, **P ≤ 0.01, ***P ≤ 0.001.

As weaker connections that did not pass our connection density thresholds (i.e., those lower than the top 2–10% of network connections) may also contribute to predicting training-related cognitive gains, we quantified module segregation [9], a metric that retains the weights of all network connections and compares within- to between-module connectivity (Fig 5A). Correlations of whole-brain network segregation and TOSL gain showed that segregation was marginally predictive of the TOSL changes in the SMART group, but not in the Control group (Fig 5B, left; Control: rho(12) = -0.22, p = 0.45; SMART: rho(13) = 0.45, p = 0.10). Separate correlations of sensory-motor and association cortex module segregation and TOSL gain showed that, in the SMART group, association cortex module segregation was significantly related to the TOSL gain (Fig 5B, left; rho(13) = 0.53, p = 0.04), but sensory-motor module segregation was not (Fig 5B, left; rho(13) = 0.25, p = 0.38). In the Control group, there was no relationship between sensory-motor or association cortex module segregation and the TOSL changes (sensory-motor: rho(12) = -0.13, p = 0.67; association cortex: rho(12) = -0.22, p = 0.45). Importantly, these results were similar when negative connections were not included in segregation calculations (i.e., only positive connections were retained; Fig 5B, middle) and when the absolute values of all connections were used to compute segregation (Fig 5B, right).

## Discussion

Our findings demonstrate that older adults with more modular brain networks at baseline showed greater improvements after cognitive training. Critically, this relationship was not present in a control group and remained significant when accounting for baseline performance on the cognitive measures that improved with training. These results are directly in line with our previous work demonstrating that TBI patients with higher brain network modularity at baseline exhibited greater improvements on executive function tasks after cognitive training [22]. We expand on these findings by demonstrating that the relationship between brain network modularity and training-related cognitive gains in healthy older adults was stronger for association cortex modules compared with sensory-motor modules. Together, these findings suggest that individuals with a more modular brain network organization measured during a task-free ‘resting-state’ prior to training are more likely to benefit from cognitive training.

Modular brain network organization is thought to support both specialized functions through communication within network modules and globally-integrated functions through communication between network modules [40]. Previous studies have provided support for the importance of this global network property by demonstrating that brain network modularity measured during a ‘resting-state’ is correlated with working memory capacity [41], predicts perception on a trial-by-trial basis [31], and is altered with varying task demands [42]. These studies suggest that modular network organization is related to both trait- and state-like aspects of cognition (e.g., working memory capacity and perceptual success, respectively). Here, we add to this previous work by showing that higher network modularity may represent an optimal brain organization for improving cognitive functioning with training. The benefits of highly modular networks have been previously demonstrated in both theoretical and empirical work. For example, computational models have shown that modular networks evolve in response to varying task goals and that this organization allows for rapid adaptation to new environments [43]. Further, individuals with higher general intelligence exhibit smaller changes in functional connectivity between a ‘resting-state’ and performance of a task, suggesting that high performing individuals have a more ‘optimal’ network organization at rest that supports more efficient changes in connectivity during task performance [44]. In the context of cognitive interventions, individuals with a more modular brain network organization may require less reconfiguration to achieve an ‘optimal’ state that allows for cognitive gains from training.

Our results also suggest that the modular organization of association cortex sub-networks may be more informative in predicting training-related gains than the modular organization of sensory-motor sub-networks. We have previously reported that SMART is associated with changes in functional connectivity of association cortex sub-networks, such as the default mode sub-network, and that these changes are associated with training-related cognitive gains [16]. This suggests that sub-networks that exhibit alterations with training may be more predictive of cognitive gains than those that do not exhibit training-related changes. Previous studies have also shown that individuals with greater segregation of association cortex modules have greater episodic memory performance [9]. In addition, association cortex modules, such as the default mode sub-network, reconfigure during working memory task performance [45–47] and, importantly, these changes are related to higher task accuracy [45]. Finally, in normal aging, association cortex modules exhibit more pronounced changes in functional connectivity compared with sensory-motor modules [9], such that association cortex modules become less ‘segregated’, or modular, with advancing age. Thus, the modular organization of association cortex sub-networks may be particularly sensitive to the aging process and important in supporting complex behaviors.

More generally, the relationship between baseline brain network modularity and training-related cognitive gains also suggests that brain network properties may be related to learning, such that individuals with a more modular brain may have a greater learning capacity and ability to benefit from training. While previous studies have shown that neural factors (e.g., frontal alpha power and striatal volume) are related to skill learning [48–51], the aspects of brain structure and function that predicted learning were variable across studies. Computational models examining the modularity of neural networks have demonstrated that more modular networks enable organisms to learn new skills without forgetting old ones [52]. Further, greater segregation of visual and motor sub-networks (i.e., more modular sub-networks) is predictive of motor learning [53] and the segregation of these sub-networks increases over the course of learning [54]. Our findings suggest that higher baseline modularity may also allow for more complex learning that is likely necessary for a cognitive intervention to be successful. Given that we have found that brain network modularity is predictive of cognitive training gains in two types of training paradigms and populations, baseline brain network modularity may provide a unifying framework that can not only be used to predict cognitive outcomes for other types of interventions, but also could be used for understanding the neural mechanisms that underlie training effects.

As brain network modularity may be a valuable biomarker that can inform the implementation of cognitive interventions, assessments of modularity could also be used to personalize interventions to maximize outcomes across individuals. For example, individuals with low baseline brain network modularity might require a longer or repeated training intervention. Although we have demonstrated that modularity is predictive of training gains in older adults and TBI patients [22], future work should also examine this relationship in healthy young adults and other patient populations to further address the generalizability of these findings. More broadly, our results also imply that brain network modularity may index individual differences in neuroplasticity. To more directly assess this, an important area of future research should be to examine the relationship between modularity and underlying training-related neural changes.

There are several limitations to the present study. First, like many cognitive intervention studies of this type, our sample size was relatively small. However, confidence in our main findings is bolstered by our previous work that found a similar relationship between baseline brain network modularity and training-related cognitive gains in TBI patients [22]. Second, while baseline modularity was predictive of changes in performance on the TOSL, a measure of gist reasoning, it was not related to training-related gains in performance on Similarities, a measure of concept abstraction, although both measures showed improvement after SMART. There are several possible explanations for this finding. The effect size of the training-related gain on Similarities was smaller than that of the TOSL. Further, in our previous study, changes in the TOSL seemed to more closely track training-related neural changes, as measured by cerebral perfusion, compared with Similarities [16]. Third, there was no active control group in this study, which limited full examination of the specificity of our results to SMART. However, another study has shown that an active control group did not exhibit training-related cognitive changes compared to SMART [23].
