# Correction: Modular Brain Network Organization Predicts Response to Cognitive Training in Older Adults

**DOI:** 10.1371/journal.pone.0174570

**Published:** 2017-03-20

**Authors:** The PLOS ONE Staff

There is an error in the penultimate sentence of the Abstract. The correct sentence is: **Trial Registration:** https://clinicaltrials.gov/ct2/show/NCT00977418. The publisher apologizes for the error.
